# CGRP alleviates lipopolysaccharide-induced ARDS inflammation via the HIF-1α signaling pathway

**DOI:** 10.1042/CS20243170

**Published:** 2025-04-09

**Authors:** Renzi Zhang, Yuhua Zhong, Qiudie Liu, Mengqi Zhang, Daoxin Wang, Sheng Li, Di Qi

**Affiliations:** Department of Respiratory and Critical Care Medicine, The Second Affiliated Hospital of Chongqing Medical University, Chongqing, China

**Keywords:** ARDS, CGRP, HIF-1α, inflammatory response, polarization of macrophages

## Abstract

Acute respiratory distress syndrome (ARDS) is an acute and severe disease with a high mortality rate. The outbreak of immune inflammation in the lung is an important pathogenic mechanism of ARDS. Notably, an imbalance in macrophage polarization is an important link in the occurrence and development of this inflammatory response. Recently, neuropeptides have been shown to regulate inflammation, but the role of neuropeptides in ARDS remains unclear. The aim of this study was to investigate the regulatory effect of calcitonin gene-related peptide (CGRP) on the inflammatory response in ARDS. We found that CGRP expression was increased in the serum of ARDS patients and in both *in vitro* and *in vivo* models of ARDS. CGRP can regulate the polarization of macrophages by targeting its receptor (receptor activity-modifying protein 1); reduce the proportion of M1 macrophages; increase the proportion of M2 macrophages; and reduce pathological injury, inflammation, oxidative stress, and apoptosis in lung tissue in LPS-induced ARDS both *in vitro* and *in vivo*. Additionally, we performed transcriptome sequencing and found that hypoxia-inducible factor-1α (HIF-1α) is involved in the above process and that CGRP can alleviate ARDS-related pathological damage, inflammation, and oxidative stress by inhibiting the HIF-1α pathway to regulate macrophage polarization balance. These results indicate that CGRP has good potential for clinical translation in the treatment of pulmonary infection in ARDS. Furthermore, this study provides new ideas for the treatment of inflammatory bursts in ARDS.

## Introduction

Acute respiratory distress syndrome (ARDS) is the most severe manifestation of acute lung injury and is caused by multiple factors that lead to the excessive elevation of alveolar capillary permeability, resulting in acute episodes of noncardiogenic edema and hypoxemia [1]. Treatment for ARDS is expensive, and its incidence and mortality rates are high, with mortality rates reaching up to 40% among patients with moderate-to-severe ARDS [2–4]. The pathogenesis of ARDS is complex, and the inflammatory response plays a key role in all stages of ARDS. Abnormal activation of macrophages, infiltration of neutrophils, and excessive secretion of proinflammatory cytokines are the causes of the inflammatory response; therefore, reducing the inflammatory response is the key to treating ARDS [5,6].

Macrophages are important cells of the innate immune system; they form the first line of defence of the alveoli against airborne particulate matter and microorganisms, and they play a key role in the inflammatory response [7–11]. There are usually two different subtypes of macrophages: classically activated (M1) macrophages and alternatively activated (M2) macrophages. The polarization of M1/M2 macrophages determines the outcome of inflammation in organs [12]. M1 and M2 macrophages play important roles in the process of pulmonary inflammation. M1 macrophages promote the occurrence and development of pulmonary inflammation, whereas M2 macrophages can secrete anti-inflammatory mediators to prevent the occurrence and development of pulmonary inflammation [13–15].

Calcitonin gene-related peptide (CGRP) is a long peptide consisting of 37 amino acids and belongs to the class of neuropeptides [16]. CGRP is synthesized and secreted by a variety of cells in addition to being produced by neurons [17]. CGRP is involved in a wide range of disorders and functions, including migraines, bone fracture healing, and neuroimmune activity [18–20]. CGRP has been shown to exert protective effects against hyperoxia-induced lung injury and allergic airway inflammation [21,22]. However, the effect of CGRP on lipopolysaccharide (LPS)-induced ARDS remains unclear.

Hypoxia-inducible factor-1 (HIF-1), which was originally identified as a protein that binds to the hypoxia response element of the erythropoietin gene under hypoxic conditions, is a nuclear transcription factor that plays a major regulatory role in the adaptive response to hypoxia [23–25]. HIF-1α is the most important isoform in the pathogenesis of inflammatory lung injury [26], but its role in LPS-induced ARDS and macrophage polarization has yet to be fully elucidated. The present study aimed to investigate the effect of CGRP on the LPS-induced inflammatory response in ARDS and the role of HIF-1α in this process.

## Overview of the study

- CGRP levels in patients with ARDS and LPS-induced *in vitro* and *in vivo* models were analyzed.
- Investigating the effect and mechanism of CGRP in LPS-induced *in vitro* and *in vivo* models.
- Transcriptome sequencing showed that HIF-1α was a downstream pathway of CGRP, and we observed whether the phenotype changed after regulating HIF-1α.
- The present study focused on lung injury, wet-to-dry (W/D) weight ratio, protein concentration in bronchoalveolar lavage fluid (BALF), inflammatory factors, oxidative stress, apoptosis, and macrophage polarization.

## Materials and methods

### Reagents

LPS (Solarbio Biotechnology, Beijing, China); CGRP (MedChemexpress, New Jersey, U.S.A.); CGRP8-37 (MedChemexpress, New Jersey, U.S.A.); dimethyloxallyl glycine (DMOG, MedChemexpress, New Jersey, U.S.A.); BCA protein assay kit (Beyotime, Shanghai, China); superoxide dismutase (SOD) reagent detection kit (Nanjing Jiancheng Bioengineering Institute, China); malondialdehyde (MDA) reagent detection kit (Nanjing Jiancheng Bioengineering Institute, China); reactive oxygen species (ROS) reagent detection kit (Beyotime, Shanghai, China); IL-1β, TNF-α, IL-6, and CGRP ELISA test kit (Youkwei Biotechnology Co., LTD, Shanghai, China); dihydroethidium (Beyotime, Shanghai, China); bovine serum albumin (Sangon Biotech, Shanghai, China); anti-CD86 antibodies (ABclonal Technology, Wuhan, China); anti-CD206 antibodies (Affinity Biosciences, Changzhou, China) antibodies; secondary antibodies (Cy3: goat-anti-rabbit, Invitrogen, Carlsbad, CA, U.S.A.); FITC (goat-anti-mouse, Abcam, Cambridge, U.K); 4′, 6-diamidino-2-phenylindole (DAPI, Aladdin, Shanghai, China); Annexin V-FITC/PI apoptosis detection kit (Beyotime, Shanghai, China); FITC anti-mouse CD86 Antibody (Biolegend, California, U.S.A.); APC anti-mouse CD206 Antibody (Biolegend, California, U.S.A.), anti-CGRP (Santa Cruz Biotechnology, Texas, U.S.A., mouse mAb); anti-myeloperoxidase (MPO) (Proteintech, Wuhan, China, rabbit mAb); anti-receptor activity modifying protein 1 (RAMP1) (Proteintech, Wuhan, China, rabbit mAb), anti-HIF-1α (Cell Signaling Technology, Boston, U.S.A., rabbit mAb), and anti-GAPDH (Proteintech, Wuhan, China, rabbit mAb); WB secondary antibodies (Proteintech, Wuhan, China); Cell Counting Kit-8 (Beyotime, Shanghai, China).

### Clinical sample collection

We enrolled 52 patients with ARDS between April 2024 and October 2024 at the Second Affiliated Hospital of Chongqing Medical University and categorized them on the day of ARDS diagnosis based on their PaO_2_/FiO_2_ ratio into mild (200＜PaO_2_/FiO_2_ ≤ 300 mmHg; *n* = 17), moderate (100＜PaO_2_/FiO_2_ ≤ 200 mmHg; *n* = 17), and severe (PaO_2_/FiO_2_ ≤ 100 mmHg; *n* = 18) based on the Berlin Definition. The serum specimens were obtained from patients with ARDS, as soon as possible after each patient met the defining criteria. Patients were followed until death in hospital or discharge home with unassisted breathing and then defined as non-survivors (*n* = 34) or survivors (*n* = 18). Demographic characteristics and clinical data including etiology of ARDS, the acute physiology and chronic health evaluation II (APACHE II) score, PaO_2_/FiO_2_, white blood cells (WBCs), PCT, and creative reaction protein concentrations were routinely inspected and recorded at diagnosis. Thereafter, comprehensive clinical outcomes are collected including the duration of mechanical ventilation, the length of RICU stay, and the length of hospital stay. To analyze the difference in CGRP levels between healthy subjects and patients with ARDS, 31 healthy subjects were recruited as controls (all participant characteristics are listed in Supplementary Tables S1 and S2). The study was approved by the Ethics Committee of the Second Affiliated Hospital of Chongqing Medical University.

### LPS-induced ARDS mouse model

SPF-grade male C57BL/6 mice weighing 18–25 g were purchased from the Experimental Animal Centre of Chongqing Medical University. The mice were housed under a 12-hour light/12-hour dark cycle before the experiments and were provided sufficient food and drinking water. The animal experimental protocols were approved by the Ethics Committee of Chongqing Medical University Animal Centre. Animal experiments were performed in the IVC2 Laboratory of the Laboratory Animal Center of Chongqing Medical University and the experimental platform of the Life Science Research Institute of Chongqing Medical University. The mice were anesthetized via an intraperitoneal injection of sodium pentobarbital (50 mg/kg), and LPS (Solarbio Biotechnology, Beijing, China) was administered via intratracheal instillation at a dose of 5 mg/kg [27]. Similarly, mice in the control group were injected with an equivalent volume of sterile phosphate-buffered saline (PBS). The mice were divided into the control, LPS, CGRP + LPS, CGRP8-37 + LPS, and DMOG + CGRP + LPS groups. In this study, CGRP (MCE, U.S.A.), CGRP8-37 (MCE, U.S.A.), and DMOG (MCE, U.S.A.) were dissolved in saline. CGRP (0.1 mg/kg [28]) and CGRP8-37 (0.5 mg/kg [29]) were given intraperitoneal injection 1 h prior to stimulation with LPS. DMOG (50 mg/kg [30]) was intraperitoneally injected 1 h before CGRP stimulation. Equal volumes of saline were administered intraperitoneally to the control and LPS groups. The mice in each group were randomly sacrificed 24 h after LPS stimulation. BALF and lung tissue were collected.

### Histological evaluation and immunohistochemistry

The lung lobes were immediately soaked in 4% paraformaldehyde and embedded in paraffin. The lung tissue was sectioned into 4-µm sections and stained with hematoxylin and eosin (H&E). The extent of lung injury was assessed by two experienced pathologists as previously described [31]. The expression of RAMP1 and MPO was detected by immunohistochemistry.

### Lung W/D weight ratio

The lung W/D weight ratio is commonly used to evaluate the degree of pulmonary edema. The right lungs of the mice were weighed and dried in a constant temperature oven at 65°C for 48  h to calculate the W/D weight ratio of the lung tissues.

### BALF total protein

The protein levels in the BALF were quantified via a BCA protein assay kit (Beyotime, Shanghai, China) according to the manufacturer’s instructions.

### ELISA

The levels of TNF-α, IL-6, and IL-1β in the BALF and RAW264.7 cell culture suspensions were determined via ELISA in accordance with the manufacturers’ instructions. Serum CGRP levels in clinical samples were also measured using the same method.

### SOD, MDA, and ROS concentration measurements

SOD, MDA, and ROS levels in lung tissue and RAW264.7 cells were detected according to the instructions of commercial kits (Nanjing Jiancheng Bioengineering Institute, China).

### TUNEL staining and ROS staining

Briefly, tissue wax blocks were used to prepare and deparaffinize 4-μm-thick sections using conventional xylene and water. The tissue was covered with a solution of proteinase K and then incubated at 37°C for 25 min. The slides were immersed in PBS (pH 7.4), subjected to three 5-min rounds of shaking on a decolorizing shaker, followed by rinsing. The nuclei were stained with DAPI and incubated in the dark at 25°C for 10 min. Next, the slides were immersed in PBS (pH 7.4) and subjected to three 5-min rounds of shaking on a decolorizing shaker. Once the slides had partially dried, they were affixed with mounting tablets that suppressed fluorescence. The samples were subsequently examined and imaged via a fluorescence microscope. Once the slides were sealed and dried, a fluorescence microscope was used to observe the quantity of positive cells (displaying red fluorescence) within the field of vision. ROS in lung tissues were detected via the superoxide-specific dye dihydroethidium (Beyotime, Shanghai, China).

### Immunofluorescence assay

The lung tissue sections were blocked with 1% bovine serum albumin (Sangon Biotech, Shanghai, China) for 15 min and incubated with anti-CD86 (diluted 1:100; ABclonal Technology, Wuhan, China) and anti-CD206 (diluted 1:100; Affinity Biosciences, Changzhou, China) antibodies overnight. The slices were subsequently incubated with the corresponding secondary antibodies (Cy3: goat-anti-rabbit, diluted 1:200, Invitrogen, Carlsbad, CA, U.S.A.; FITC: goat-anti-mouse, diluted 1:200, Abcam, Cambridge, U.K), followed by counterstaining with DAPI (Aladdin, Shanghai, China). A fluorescence microscope (Olympus, Tokyo, Japan) was used to capture stained images.

### Cell culture and treatment

RAW264.7 cells (SC-6003, ATCC) were cultured in DMEM (Gibco, U.S.A.) supplemented with 10% foetal bovine serum (Gibco, U.S.A.) at 37°C with 5% CO2. To determine the effect of CGRP on the viability of RAW264.7 cells, different concentrations of CGRP (1, 10, 100, or 1000 nmol/l [32]) were added to the RAW264.7 cells, which were cultured for 1 h; then, 1 µg/ml LPS was added to the RAW264.7 cells and the cells were cultured for 24 h. CGRP8-37 (1000 nmol/l [33]) was used to inhibit CGRP. To examine the effects of the HIF-1α signaling pathway on the protective effect of CGRP on LPS-induced ARDS, the cells were treated with 1  mmol/l [34] DMOG (a HIF-1α activator) for 1  h prior to CGRP treatment. Finally, the cell samples and cell suspensions were collected.

### Apoptosis assay by flow cytometry

An Annexin V-FITC/PI apoptosis detection kit was used to determine the apoptosis ratio *in vitro*. Briefly, the cells were collected, resuspended, and incubated with FITC-conjugated Annexin V (5 µl) and PI (5 µl) for 15–20 min. The percentage of apoptotic cells was analyzed via a CytoFLEX flow cytometer (Beckman Coulter, Inc., Georgia, U.S.A.).

### Flow cytometry analysis of the macrophage phenotype

*In vitro*, RAW264.7 cells were cultured as previously described, and the expression of CD86+ and CD206+ was detected. To characterize the M1 phenotype, the cells were labeled with a CD86 (BioLegend, U.S.A.) antibody. To characterize the M2 phenotype, the cells were labeled with a CD206 (BioLegend, U.S.A.) antibody. The data were subsequently analyzed via FlwJo software (Ashland, OR, U.S.A.).

### Western blot

Proteins were extracted from lung tissues and cells as previously described [35]. Equal amounts of proteins were loaded onto 10% sodium dodecyl sulfate–polyacrylamide gels and transferred onto polyvinylidene fluoride membranes. The membranes were blocked with 5% nonfat milk in TBST for 1–2 h at 37°C and incubated overnight at 4°C with primary antibodies against CGRP (diluted 1:200; Santa Cruz Biotechnology, U.S.A.), RAMP1 (diluted 1:1000; Proteintech, China), HIF-1α (diluted 1:1000; CST, U.S.A.), and GAPDH (diluted 1:5000; Proteintech, China). Then, the membranes were incubated with the corresponding secondary antibodies (diluted 1:3000; Proteintech, China) at room temperature for 1 h. Finally, the protein bands were visualized via a ChemiDoc Touch Imaging System (Bio-Rad, CA, U.S.A.).

### Cell viability assay

A Cell Counting Kit-8 (CCK8, Beyotime, China) was used to detect the viability of the samples. It was used in conjunction with the instructions provided by the manufacturer for detecting cell viability in the sample. Overnight incubation was performed with 5 × 10^3^ cells per well in 96-well plates. Each well of the plate was subsequently filled with 10 μl of CCK-8 solution and treated for 2 h. The absorbance of each well was then measured at 450 nm.

### Statistical analysis

Statistical analysis was performed via GraphPad Prism software, and the data are presented as the means ± SDs. Student’s *t*-test was used to assess differences between two groups, whereas one-way ANOVA was used for comparisons involving three or more groups. A value of *P* < 0.05 was considered statistically significant.

## Results

### CGRP expression levels were elevated in ARDS and inversely proportional to the severity of ARDS

**Figure 1:**
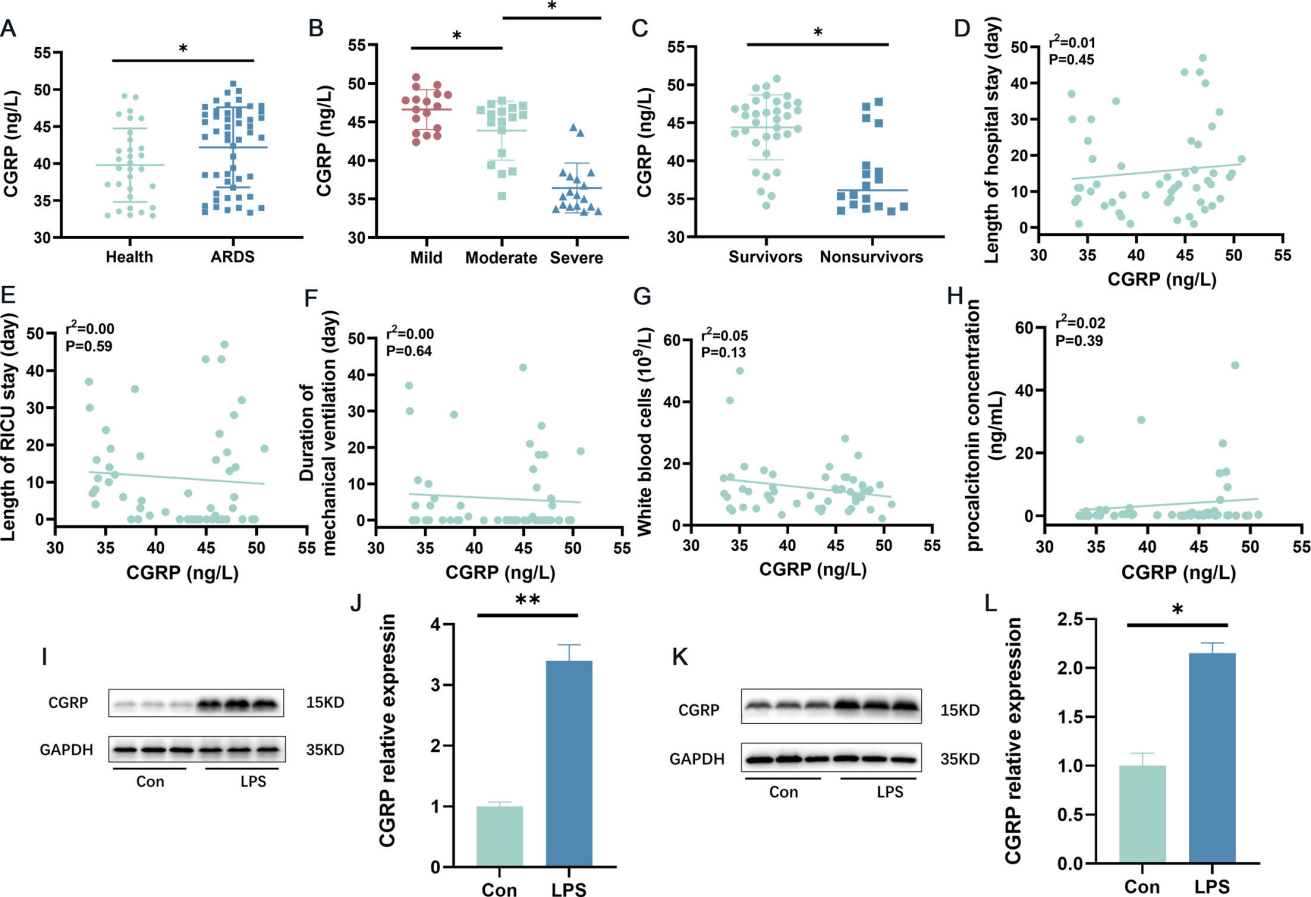
CGRP expression levels are elevated in patients with ARDS and in experimental models of ARDS. (**A**) ELISA was used to quantitatively evaluate blood CGRP levels in ARDS patients and controls (controls = 31, ARDS patients = 52).** (B) **Serum CGRP levels in patients with mild-to-moderate ARDS. (**C) **CGRP content and survival of ARDS patients. (**D–H) **Trend plots of CGRP content and length of hospital stay, RICU stay, mechanical ventilation time, total white blood cell count, and procalcitonin in ARDS patients. (**I,J) **Western blot (WB) analysis of CGRP protein levels in ARDS mice and controls (*n* = 3). (**K,L) **WB analysis of CGRP expression in RAW264.7 cells treated with LPS (1 µg/ml) and control cells (*n* = 3). *P < 0.05, **P < 0.01. ARDS, acute respiratory distress syndrome; CGRP, calcitonin gene-related peptide.

We evaluated blood CGRP expression in ARDS patients and controls via ELISA and found that CGRP expression levels were elevated in ARDS patients (Figure 1A). We also investigated the relationships between CGRP expression level and ARDS severity, survival, length of hospital stays, RICU length of stay, duration of mechanical ventilation, WBC count, and procalcitonin (PCT) content. The level of CGRP expression was inversely proportional to the severity of ARDS: patients with high CGRP levels had less severe disease, while those with low CGRP levels had more severe disease (Figure 1B). In addition, the level of CGRP expression was proportional to the survival rate of patients with ARDS, with lower levels of CGRP associated with poorer survival (Figure 1C). In ARDS patients, no significant correlations were observed between CGRP expression levels and length of hospital stay, RICU lengths of stay, durations of mechanical ventilation, total white blood cell counts, or PCT levels (Figure 1D–H). In an *in vivo* model of LPS-induced ARDS, Western blot (WB) analysis revealed that the protein levels of CGRP were increased compared with those in normal mice (Figure 1I and J). In addition, we selected the RAW264.7 macrophage line to evaluate the CGRP expression in the LPS-induced *in vitro* model, and WB experiments revealed that the CGRP content was elevated in the LPS-induced *in vitro* model compared with that in control cells (Figure 1K and L). These results suggest that CGRP may play an important role in the pathogenesis and treatment of ARDS.

### CGRP alleviated LPS-induced lung injury *in vivo*

**Figure 2:**
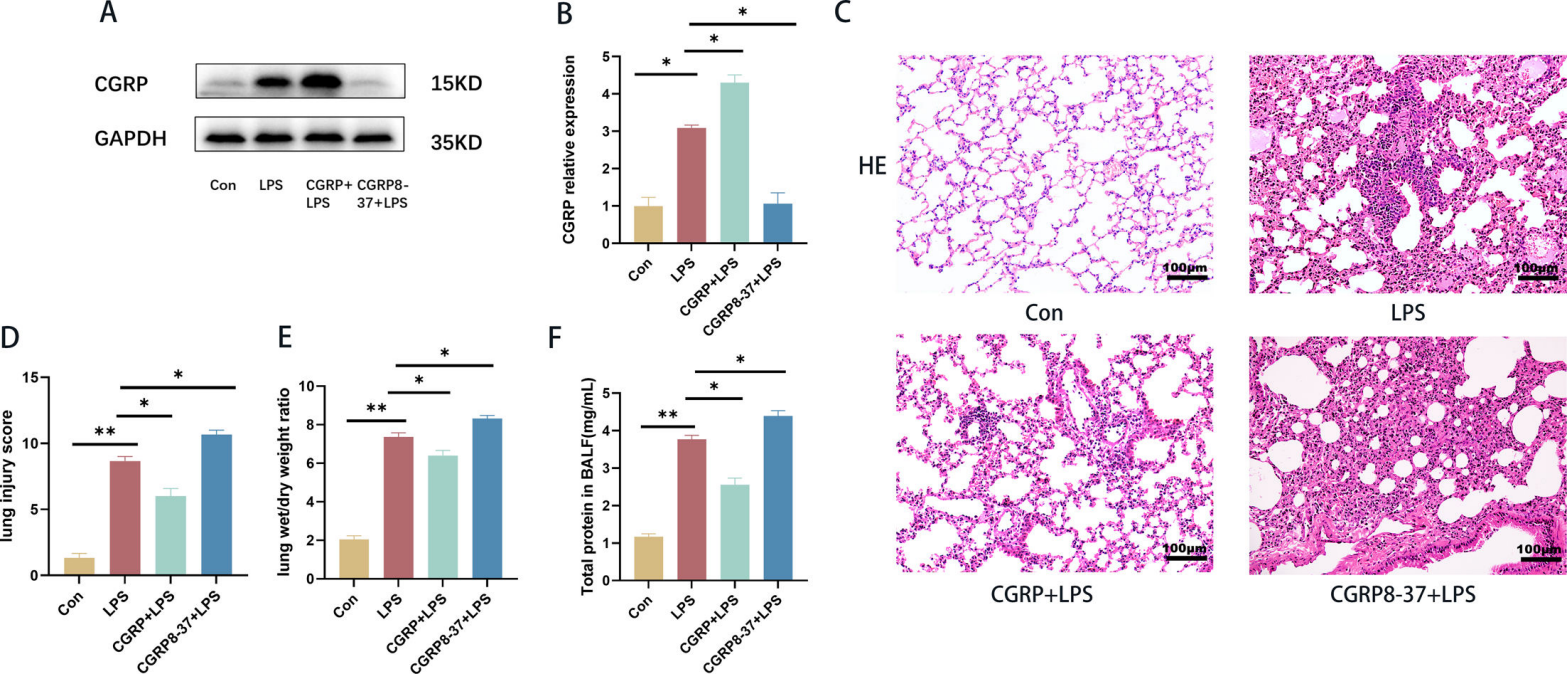
CGRP attenuated LPS-induced lung injury. (**A,B**) CGRP expression in the lung. (**C) **H&E staining (scale bar = 100 μm). (**D) **Lung injury scores. (**E) **Lung W/D weight ratio. (**F) **Total protein levels in the BALF. *P < 0.05, **P < 0.01. BALF, bronchoalveolar lavage fluid; CGRP, calcitonin gene-related peptide; H&E, hematoxylin and eosin; LPS, lipopolysaccharide; W/D, wet-to-dry weight ratio.

We evaluated the therapeutic effect of CGRP on lung inflammation and injury via the intraperitoneal injection of CGRP and CGRP8-37, a specific inhibitor of CGRP. CGRP expression is shown in the figure (Figure 2A and B). Compared with the control group, the LPS group presented more severe inflammatory cell infiltration, alveolar hemorrhage, interstitial edema, and diffuse alveolar damage. However, these symptoms in the CGRP peptide group were alleviated compared with those in the LPS group, and the symptoms in the CGRP inhibitor group were aggravated compared with those in the LPS group (Figure 2C). Compared with those in the normal group, the lung injury score, lung W/D weight ratio, and total protein concentration were significantly greater in the LPS group. The administration of CGRP peptides significantly inhibited these changes, whereas the administration of CGRP inhibitors promoted these changes (Figure 2D–FF). Taken together, these data suggest that CGRP can attenuate LPS-induced lung injury in mice.

### CGRP alleviated LPS-induced oxidative stress and the inflammatory response

**Figure 3:**
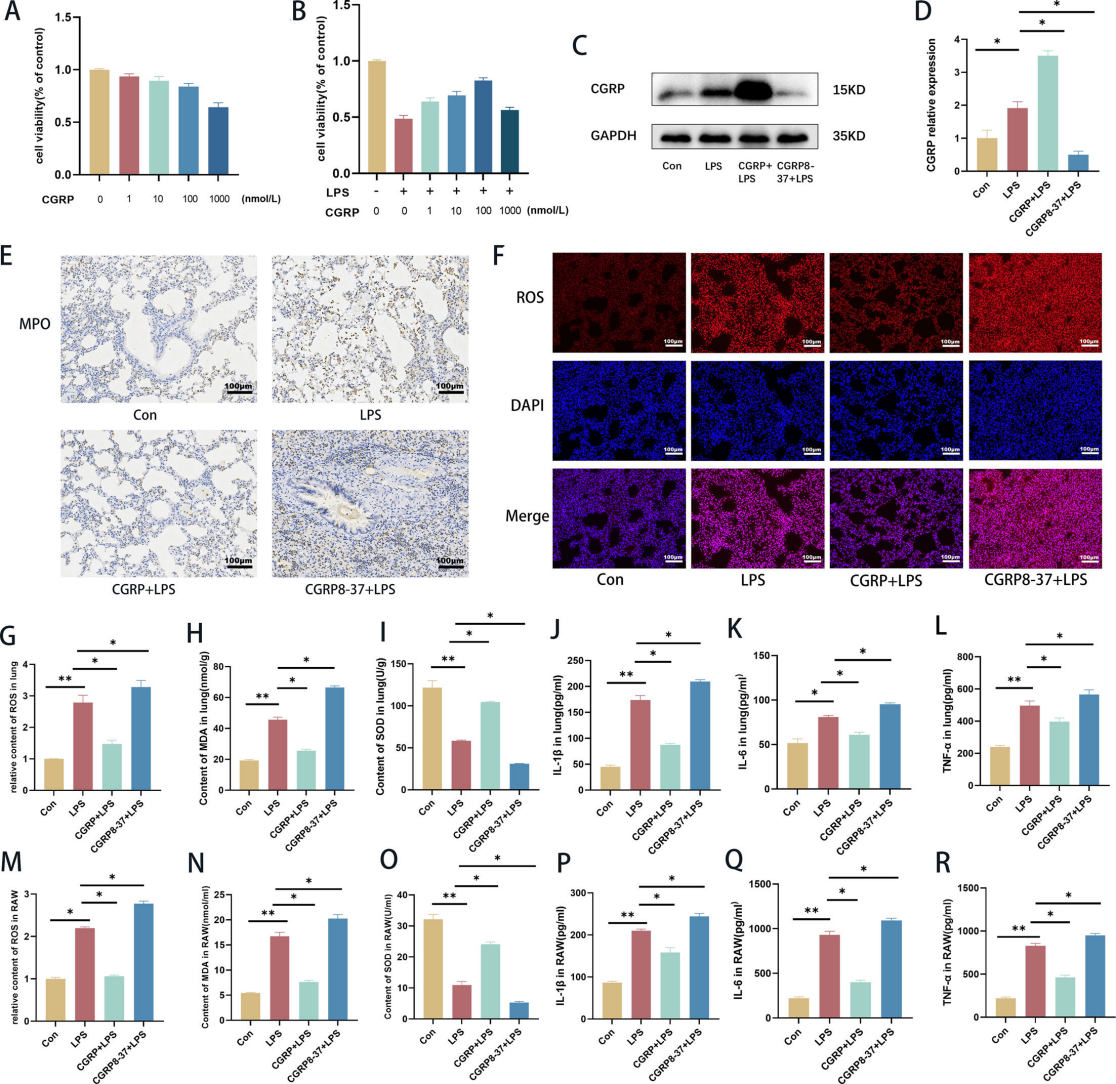
CGRP attenuates LPS-induced oxidative stress and inflammation. (**A**) RAW264.7 cells were treated with different concentrations of CGRP for 24 h, and the cell viability was calculated. (**B) **RAW264.7 cells were treated with different concentrations of CGRP after LPS stimulation for 24 h, and the cell viability was calculated.** (C,D) **CGRP expression in RAW264.7 cells. (**E) **MPO expression in lung tissue (scale bar = 100 μm). (**F) **ROS staining in lung tissue (scale bar = 100 μm). (**G–I**) ROS, MDA, and SOD levels in the lungs. (**J–L**) Concentrations of IL-1β, IL-6, and TNF-α in the lungs. (**M–O**) ROS, MDA, and SOD contents in RAW264.7 cells. (**P–R**) Concentrations of IL-1β, IL-6, and TNF-α in RAW264.7 cells. The data are presented as the mean ± S.D. *P < 0.05, **P < 0.01. CGRP, calcitonin gene-related peptide; LPS, lipopolysaccharide; MDA, malondialdehyde; MPO, myeloperoxidase; ROS, reactive oxygen species; SOD, superoxide dismutase.

A CCK-8 assay was used to detect the protective effect of CGRP on LPS-induced RAW264.7 cell viability. RAW264.7 cells were treated with different concentrations of CGRP (0, 1, 10, 100, or 1000 nmol/l) to determine the optimal concentration of CGRP. According to the CCK-8 release rate, CGRP at concentrations other than 1000 nmol/l was not significantly toxic to the cells (Figure 3A), and 100 nmol/l CGRP was the best concentration for protecting the cells (Figure 3B). Therefore, 100 nmol/l was chosen as the concentration of CGRP to further validate our findings. Oxidative damage and inflammatory factors play important roles in LPS-induced ARDS. We examined CGRP expression *in vitro* (Figure 3C and D). The contents of ROS, MDA, SOD, IL-1β, IL-6, and TNF-α in the lung tissue of ARDS mice were detected via a microplate reader. The expression of MPO in the lung tissue was detected via immunohistochemistry. ROS staining was used to detect its expression in the lung. The contents of ROS, MDA, SOD, IL-1β, IL-6, and TNF-α in the RAW264.7 cell inflammation model were detected via the same method. *In vivo*, treatment with the CGRP peptide significantly reduced LPS-induced ROS, MDA, and MPO production and increased the SOD content compared with those in the LPS group. However, the administration of a CGRP inhibitor increased the production of ROS, MDA, and MPO and decreased the content of SOD. Compared with that in the LPS group, the secretion of proinflammatory factors (IL-1β, IL-6, and TNF-α) was decreased in the CGRP group, while the secretion of proinflammatory factors was increased in the CGRP inhibitor group (Figure 3E–L). The same results were obtained in the *in vitro* experiments. Compared with those in the LPS group, the levels of ROS, MDA, SOD, IL-1β, IL-6, and TNF-α were lower, and the level of SOD was greater in the CGRP group. Compared with those in the LPS group, the levels of ROS, MDA, SOD, and inflammatory factors were increased, and the level of SOD was decreased in the CGRP inhibitor group (Figure 3M–R). These results suggest that CGRP attenuates LPS-induced oxidative stress and inflammation in ARDS.

### CGRP attenuated LPS-induced apoptosis

**Figure 4:**
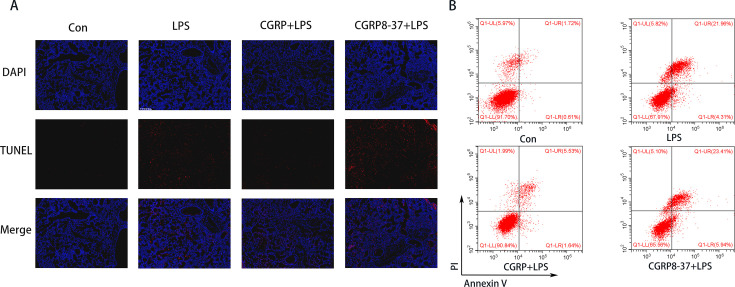
CGRP attenuated LPS-induced apoptosis. (**A**) TUNEL staining of the lung (blue = nucleus, red = TUNEL, scale bar = 200 μm). (**B) **The percentage of apoptotic RAW264.7 cells was detected via flow cytometry. CGRP, calcitonin gene-related peptide; LPS, lipopolysaccharide.

Apoptosis also plays an important role in the progression of ARDS. Therefore, LPS-induced apoptosis was also investigated. *In vivo* lung tissue apoptosis staining revealed that, compared with the LPS group, the CGRP group exhibited reduced apoptosis of lung tissue cells, whereas the CGRP inhibitor group exhibited aggravated apoptosis of lung tissue cells (Figure 4A). *In vitro* flow cytometry also revealed that, compared with the LPS group, the CGRP group exhibited significantly reduced cell apoptosis, whereas the CGRP inhibitor group presented significantly increased cell apoptosis (Figure 4B).

### CGRP decreased the proportion of M1 macrophages and increased the proportion of M2 macrophages in both *in vitro* and *in vivo* models of ARDS

**Figure 5:**
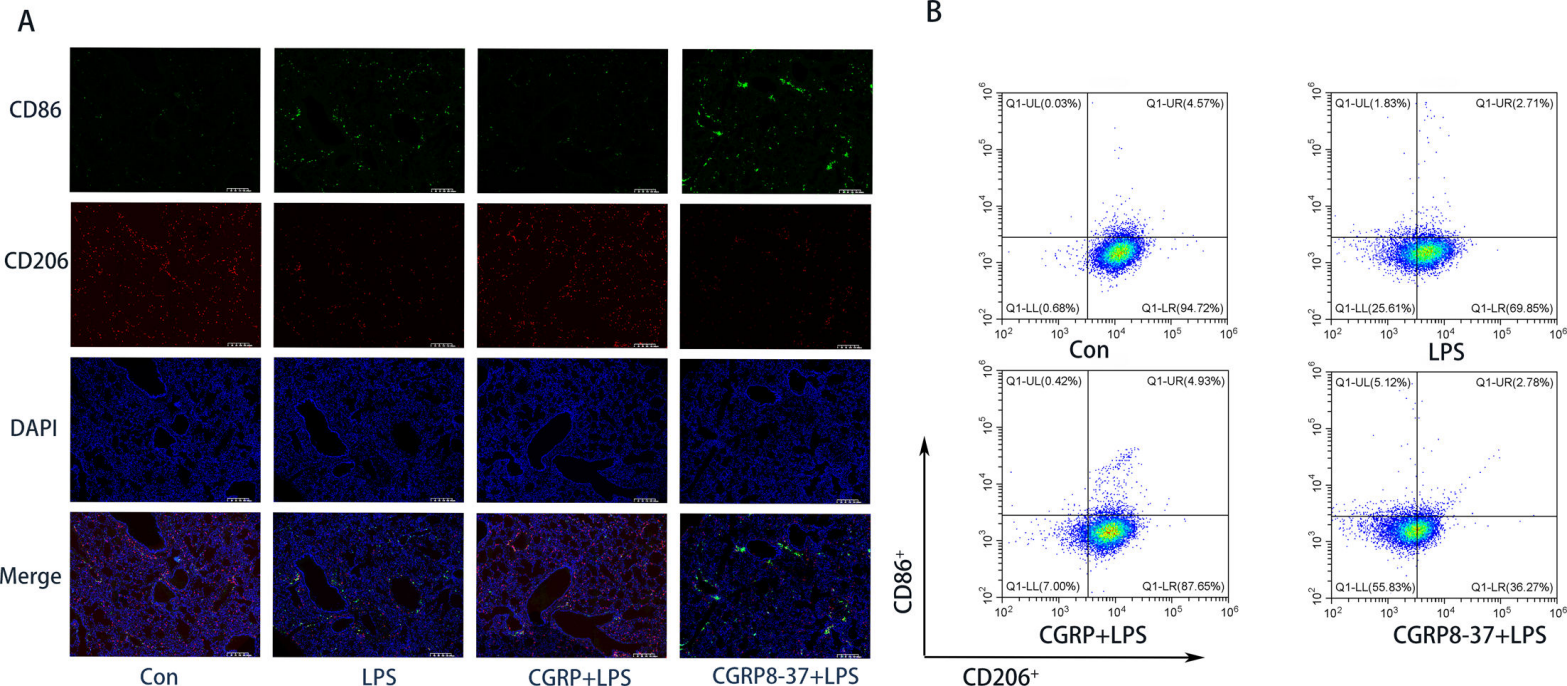
CGRP regulates the polarization balance of M1 (CD86)/M2 (CD206) macrophages in LPS-induced ARDS. (**A**) Immunofluorescence in the lung (blue = nucleus, red = CD206, green = CD86, scale bar = 200 μm). (**B) **Flow cytometry was used to detect the proportion of M1 (CD86)/M2 (CD206) macrophages among RAW264.7 cells. ARDS, acute respiratory distress syndrome; CGRP, calcitonin gene-related peptide; LPS, lipopolysaccharide.

M1 macrophages promote the occurrence and development of inflammation, which is associated with high levels of IL-1β, IL-6, and TNF-α secretion. CD86 is the main surface molecular marker of M1 macrophages [36,37]. M2 macrophages play a role in inhibiting tissue and cellular inflammation and promoting tissue repair and angiogenesis. The main surface molecular markers of M2 macrophages include CD206 and Arg-1 [38–40]. Lung immunofluorescence assays revealed that the CGRP treatment inhibited the proportion of M1 macrophages (CD86) and increased the proportion of M2 macrophages in the lungs of the LPS-induced model mice. The CGRP inhibitor increased the number of M1 macrophages and decreased the number of M2 macrophages in the lungs of the LPS-induced model mice (Figure 5A). In addition, 100 nM CGRP was added to RAW264.7 cells 1 h before LPS treatment. The flow cytometry results revealed that the CGRP treatment reduced the proportion of M1-type macrophages and increased the proportion of M2-type macrophages in RAW264.7 cells induced by LPS. The CGRP inhibitor had showed opposite effect (Figure 5B). The results showed that CGRP could regulate the balance of M1/M2 macrophages in LPS-induced ARDS models *in vitro* and *in vivo*, thereby regulating the pulmonary inflammatory response in ARDS.

### RAMP1/HIF-1α expression in the LPS-induced ARDS model

**Figure 6:**
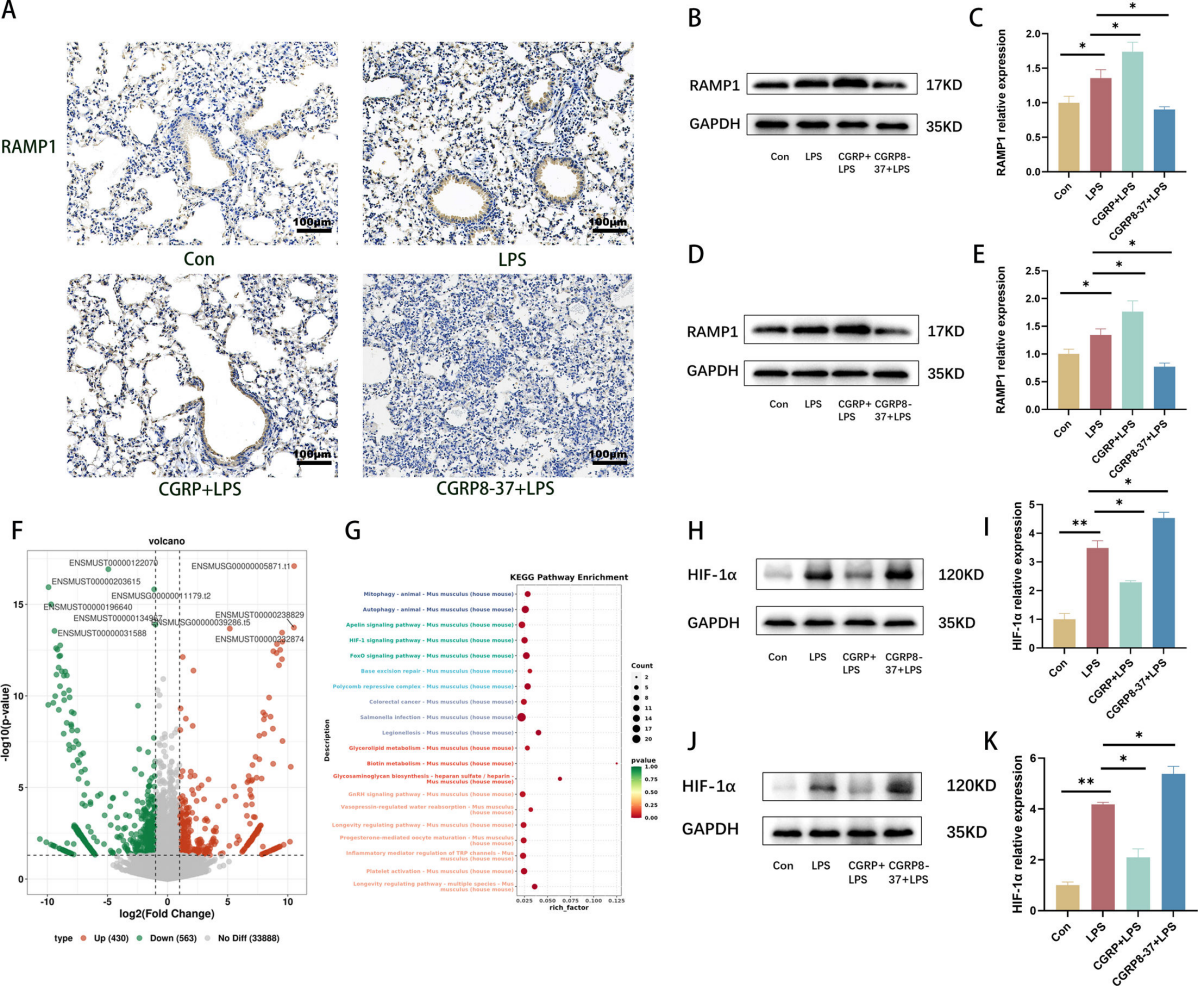
Expression of RAMP1 and CGRP in the ARDS model. (**A**) RAMP1 expression in the lung (scale bar = 100 μm).** (B,C) **Protein expression of RAMP1 in the lung.** (D,E) **Protein expression of RAMP1 in RAW264.7 cells. (**F,G) **Volcano plot showing the differentially expressed genes identified by RNA-seq in the LPS and CGRP + LPS groups of RAW264.7 cells. (**H,I) **Protein expression of HIF-1α in the lung.** (J,K) **Protein expression of HIF-1α in RAW264.7 cells. The data are presented as the mean ± S.D. *P < 0.05, **P < 0.01. ARDS, acute respiratory distress syndrome; CGRP, calcitonin gene-related peptide; LPS, lipopolysaccharide; RAMP1, receptor activity-modifying protein 1.

CGRP signals are sent to cells through a heterodimeric receptor composed of the calcitonin receptor-like receptor, RAMP1, and the cytoplasmic association of receptor component protein; these components regulate various signaling activities in cells [41]. As an important component of the CGRP receptor, RAMP1 enables immune cells to effectively sense neuronal modulation. Therefore, we also investigated the expression of RAMP1 in this study [42]. To further investigate the underlying mechanism of CGRP action, LPS and CGRP + LPS-treated macrophages were collected for RNA-sequencing. Immunohistochemistry and WB revealed that the expression of RAMP1 was increased in the LPS group, further increased in the CGRP group, and decreased in the CGRP inhibitor group compared with the LPS group, which was consistent with the changes in CGRP (Figure 6A–E). We identified 563 genes with decreased expression after CGRP peptide administration (Figure 6F and G). Among these 563 genes, we focused on the metabolic and inflammatory response-related gene HIF-1α. RNA sequencing revealed that HIF-1α expression was lower in the CGRP group than in the LPS group in the *in vitro* model. WB analysis also revealed that the expression of HIF-1α was increased in the LPS group, decreased in the CGRP group, and further increased in the CGRP inhibitor group compared with that in the LPS group (Figure 6H–K). Therefore, we hypothesized that HIF-1α pathway may be involved in the mechanism of CGRP-alleviating ARDS.

### CGRP ameliorates LPS-induced ARDS through the HIF-1α pathway

**Figure 7:**
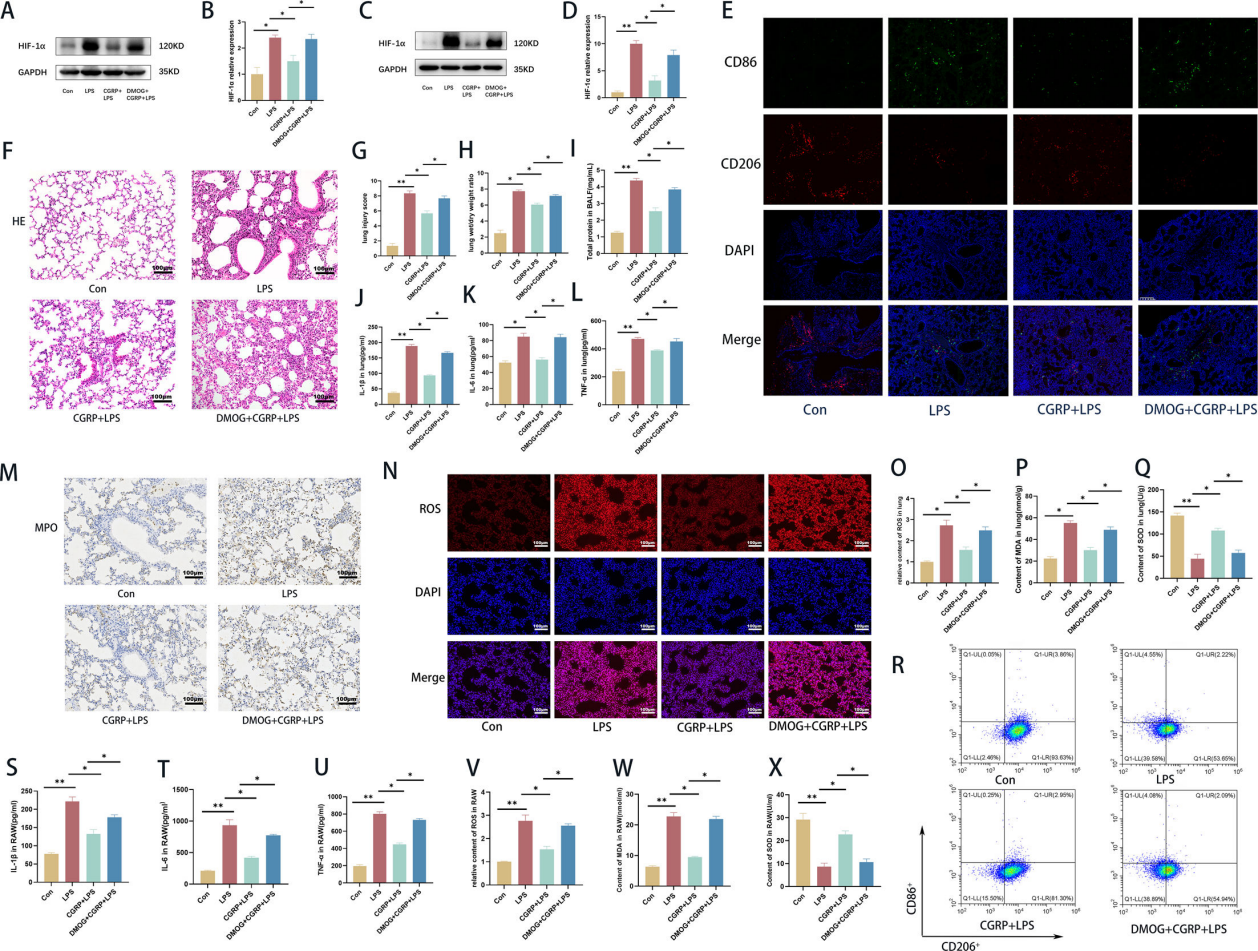
CGRP alleviates LPS-induced ARDS through the HIF-1α pathway. (**A,B**) Protein expression of HIF-1α in the lung. (**C,D) **Protein expression of HIF-1α in RAW264.7 cells. (**E) **Immunofluorescence in the lung (blue = nucleus, red = CD206, green = CD86, scale bar = 200 μm).** (F) **H&E staining (scale bar = 100 μm). (**G) **Lung injury scores.** (H) **Lung W/D weight ratio. (**I) **Total protein levels in the BALF. (**J–L**) Concentrations of IL-1β, IL-6, and TNF-α in the lungs. (**M,N) **MPO and ROS levels in the lungs (scale bar = 100 μm). (**O–Q**) ROS, MDA, and SOD contents in the lungs.** (R) **Flow cytometry was used to detect the proportion of M1 (CD86)/M2 (CD206) macrophages among the RAW264.7 cells. (**S–U**) Concentrations of IL-1β, IL-6 and TNF-α in RAW264.7 cells. (**V–X**) ROS, MDA, and SOD contents in RAW264.7 cells. The data are presented as the mean ± S.D. *P < 0.05, **P < 0.01. CGRP, calcitonin gene-related peptide; H&E, hematoxylin and eosin; HIF-1α**,** hypoxia-inducible factor-1α; LPS, lipopolysaccharide; MDA, malondialdehyde; MPO, myeloperoxidase; ROS, reactive oxygen species; SOD, superoxide dismutase.

CGRP regulates macrophage polarization and LPS-induced ARDS through the HIF-1α pathway. We used the HIF-1α-specific activator DMOG to deactivate the HIF-1α pathway. WB was used to detect the expression of HIF-1α *in vitro* and *in vivo* (Figure 7A–D). In an *in vivo* model of ARDS, a lung immunofluorescence assay revealed that the number of M1 macrophages decreased, while the number of M2 macrophages increased in the CGRP group. DMOG reversed the regulatory effect of CGRP on macrophage polarization, such that the number of M1 macrophages increased, while the number of M2 macrophages decreased (Figure 7E). H&E staining revealed that, compared with the CGRP group, the DMOG group presented more severe lung injury (Figure 7F and G). In addition, the W/D weight ratio of the lungs and protein concentration in the BALF were greater in the DMOG group than in the CGRP group (Figure 7H and I). ELISAs revealed that the levels of IL-1β, IL-6, and TNF-α were decreased in the CGRP group but were increased in the MDOG group (Figure 7J–L). In addition, the effect of CGRP on oxidative stress through the HIF-1α pathway was investigated. Immunohistochemistry revealed that the decreases in MPO and ROS levels increased again after HIF-1α activation (Figure 7M and N). The contents of ROS, MDA, and SOD were also reversed in the CGRP group after HIF-1α activation (Figure 7O–Q). *In vitro* flow cytometry revealed the same results, and the regulatory effect of CGRP on macrophage polarization was reversed by DMOG (Figure 7R). The levels of IL-1β, IL-6, and TNF-α in the DMOG group were significantly greater than those in the CGRP group *in vitro* (Figure 7S–U). The levels of ROS and MDA in the DMOG group were greater than those in the CGRP group, whereas the level of SOD was lower in the DMOG group (Figure 7V–X). Overall, we demonstrated that CGRP regulated the polarization balance of macrophages through the HIF-1α pathway and alleviated the LPS-induced inflammatory response and oxidative stress in ARDS *in vitro* and *in vivo*.

## Discussion

In recent decades, there has been considerable progress in understanding the epidemiology, pathogenesis, and pathophysiology of ARDS. Furthermore, randomized trials have been conducted to examine the optimization of mechanical ventilation and fluid therapy for ARDS, thus yielding improved clinical outcomes. Despite the progress that has been made in the supportive care of ARDS patients, such as appropriate antimicrobial therapy, early enteral nutrition, and the prevention of venous thromboembolism and gastrointestinal ulcers, effective medical treatments for ARDS have yet to be identified [43,44]. In recent years, the interaction between neuroimmunity has become a research hotspot in the fields of neurobiology and immune-related inflammation. As a new mechanism that regulates the inflammatory response in various diseases, the role of neuroimmunity deserves further study. The nervous system can receive various stimuli and transmit signals to surrounding organs in the form of neurotransmitters and/or neuropeptides through specific receptors, thereby regulating the inflammatory response of the surrounding organs [45]. Neuroimmune interactions are among the driving factors of a variety of pulmonary inflammatory diseases, including ARDS. Therefore, the modulation of neuroimmune interactions is a promising therapeutic target for ARDS. Exploring the effects of the peripheral nervous system and its neurotransmitters and/or neuropeptides on the pulmonary immune microenvironment will help in the identification of effective drugs for the treatment of ARDS [46].

The resolution of inflammation requires the regulation of proinflammatory and anti-inflammatory mediators and the transformation of macrophage phenotype [47]. Many preclinical studies have shown that targeting macrophage polarization is an effective and promising treatment for ARDS, but the effects of polypeptide proteins associated with the nervous system on macrophage polarization and lung inflammation have yet to be fully elucidated [48,49]. Some scholars have noted that CGRP may be one of the neuropeptides involved in the host inflammatory response [50,51]. In our study, we found that CGRP expression was increased in ARDS patients and ARDS models both *in vitro* and *in vivo*. CGRP is a neuropeptide that is homologous to calcitonin gene, derived from the selective cleavage of the primary transcription product of the calcitonin gene. Consequently, its biological activity is nearly identical to that of calcitonin, and it plays a crucial regulatory role in inflammatory diseases [52]. CGRP levels were increased in response to acute stress stimulation of ARDS but were negatively correlated with the severity of ARDS, indicating a protective regulatory effect of CGRP in the pathophysiology of ARDS. This was consistent with the results of our collected patient samples, where CGRP expression was increased in patients with mild ARDS, while it was decreased in those with severe ARDS. The conclusion of Barbosa et al. is same as that of our study: CGRP levels in critically ill COVID-19 patients increased early in the illness in SARS-CoV-2-positive patients and gradually returned to baseline levels as the virus cleared and the disease improved [53,54]. CGRP was shown to alleviate lung injury, inflammation, oxidative stress, and apoptosis in LPS-induced ARDS through the CGRP receptor (RAMP1), inhibit the classical activation of macrophages, and promote the alternative activation of macrophages.

Previous studies have shown that the modulation of the HIF-1α pathway can reduce inflammatory responses in mouse models of inflammatory bowel disease [55]. Sang et al. reported that the modulation of the HIF-1α pathway alleviated a model of inflammation-induced liver injury [56]. However, it remains unclear whether CGRP regulates macrophage polarization and alleviates LPS-induced ARDS through the HIF-1α pathway. First, we found that HIF-1α activation was suppressed by the administration of the CGRP peptide but enhanced by the administration of a CGRP inhibitor. Subsequently, HIF-1α was activated by a HIF-1α-specific activator (DMOG). Compared with the CGRP group, the DMOG group presented more severe lung injury, inflammatory responses, and oxidative stress. In addition, the number of M1 macrophages decreased, and the number of M2 macrophages increased. Taken together, our findings suggest that CGRP alleviates LPS-induced ARDS by polarizing macrophages from the proinflammatory M1 phenotype to the anti-inflammatory M2 phenotype through HIF-1α signaling. These findings may provide a novel strategy for the treatment and prevention of ARDS. In addition, as a neuropeptide that regulates lung immunity, CGRP is widely expressed in the nervous system and can be released by some lung neurons. The mechanism of action of these neurons in pulmonary immunity, especially in ARDS, remains unclear and is a weakness of this study. Further research is necessary to determine whether these neurons can directly regulate lung inflammation, maintain lung tissue homeostasis, and have a direct effect on ARDS.

In conclusion, the neuropeptide CGRP plays an important role in LPS-induced ARDS. CGRP binds to the CGRP receptor and regulates the transition of the macrophage phenotype from M1 to M2 through the HIF-1α signaling pathway, thereby reducing lung injury, inflammation, and oxidative stress. Our study suggests that neuroimmune dialogue plays a role in maintaining tissue homeostasis by promoting resolution of inflammation.

**Clinical Perspectives**

- Acute respiratory distress syndrome (ARDS) can lead to death and affect the quality of life of patients. The expression of calcitonin gene-related peptide (CGRP) is increased in ARDS patients.
- In clinical study samples, CGRP expression in lung tissue correlated with the ARDS severity, length of hospital stay, duration of mechanical ventilation, and levels of inflammatory markers. In addition, CGRP can reduce lung tissue injury and inflammatory response in ARDS *in vivo* and *in vitro*.
- With the lack of effective pharmacological treatment for ARDS, our study helps to validate CGRP as a new hope for lung protection in ARDS.

## Supplementary material

Supplementary Table S1

Supplementary Table S2

## Data Availability

All data included in this study are available upon request by contact with the corresponding author.

## Abbreviations

ARDS: acute respiratory distress syndrome
BALF: bronchoalveolar lavage fluid
CGRP: calcitonin gene-related peptide
DAPI: 4’,6-diamidino-2-phenylindole
DMOG: dimethyloxallyl glycine
H&E: hematoxylin and eosin
HIF-1α: hypoxia-inducible factor-1α
LPS: lipopolysaccharide
MDA: malondialdehyde
MPO: myeloperoxidase
PCT: procalcitonin
RAMP1: receptor activity-modifying protein 1
ROS: reactive oxygen species
SOD: superoxide dismutates
WB: Western blot
WBC: white blood cell count
