# Supplementary material for: CGRP alleviates lipopolysaccharide-induced ARDS inflammation via the HIF-1α signaling pathway
Source: Clin Sci (Lond). 2025 Apr 9;139(7):373–87. doi: 10.1042/CS20243170 (PMC12204009; doi:10.1042/CS20243170)
Supplement: Supplementary Table S1 [file CS-139-07-CS20243170-s001.docx]

**Table S1. Demographic characteristics of all participants**

| variable | Healthy control (n=31) | ARDS (n=52) |
| --- | --- | --- |
| WBC (10^9^/L) | 6.868±2.651 | 12.08±8.493 * |
| PCT (ng/ml) | 0.1385±0.1719 | 3.640±9.064 * |
| CGRP (ng/L) | 39.78±4.963 | 42.19±5.412 * |

*: *P*<0.05.
