# Supplementary material for: CGRP alleviates lipopolysaccharide-induced ARDS inflammation via the HIF-1α signaling pathway
Source: Clin Sci (Lond). 2025 Apr 9;139(7):373–87. doi: 10.1042/CS20243170 (PMC12204009; doi:10.1042/CS20243170)
Supplement: Supplementary Table S2 [file CS-139-07-CS20243170-s002.docx]

**Table S2. Demographic characteristics of ARDS patients**

| Variable | Hospital survivors  (n=34) | Hospital non-survivor  (n=18) |
| --- | --- | --- |
| Age (year) | 66.56±10.75 | 70.56±10.06 |
| Gender (n, %) |  |  |
| Male | 23(44.2) | 15(28.8) |
| Femal | 11(21.2) | 3(5.8) |
| Pathogeny | severe community-acquired pneumonia | severe community-acquired pneumonia |
| APACHE II score | 20.15±8.143 | 29.00±8.738 * |
| PaO_2_/ FiO_2_ | 193.8±75.84 | 87.89±31.96 * |
| WBC (10^9^/L) | 10.94±5.596 | 14.24±12.17 |
| PCT (ng/ml) | 3.518±9.359 | 3.869±8.737 |
| CGRP (ng/L) | 44.39±4.269 | 38.03±4.951 * |
| ARDS severity (n, %) |  |  |
| Mild | 17 (32.6) | 0 (0) |
| Moderate | 10 (19.2) | 7(13.4) |
| Severe | 7 (13.4) | 11 (21.1） |
| Length of hospital stay (day) | 14.35±11.26 | 17.72±13.28 |
| Length of ICU stay (day) | 9.588±13.14 | 13.94±13.45 |
| Length of mechanical ventilation stay (day) | 3.882±7.892 | 10.11±13.78 * |

*: *P*<0.05.
